# Theoretical Study on Zearalenol Compounds Binding with Wild Type Zearalenone Hydrolase and V153H Mutant

**DOI:** 10.3390/ijms19092808

**Published:** 2018-09-18

**Authors:** Ye Liu, Youzhong Wan, Jingxuan Zhu, Zhengfei Yu, Xiaopian Tian, Jiarui Han, Zuoming Zhang, Weiwei Han

**Affiliations:** Key Laboratory for Molecular Enzymology and Engineering of the Ministry of Education, National Engineering Laboratory of AIDS Vaccine, College of Life Science, Jilin University, Changchun 130023, China; lye16@mails.jlu.edu.cn (Y.L.); wanyouzhong@jlu.edu.cn (Y.W.); zhujx15@mails.jlu.edu.cn (J.Z.); yuzf16@mails.jlu.edu.cn (Z.Y.); tianxp1314@mails.jlu.edu.cn (X.T.); jrhan17@mails.jlu.edu.cn (J.H.); zmzhang@jlu.edu.cn (Z.Z.)

**Keywords:** zearalenone hydrolase, molecular dynamics simulation, protein network analysis, alanine scanning, conformational change

## Abstract

Zearalenone hydrolase (ZHD) is the only reported α/β-hydrolase that can detoxify zearalenone (ZEN). ZHD has demonstrated its potential as a treatment for ZEN contamination that will not result in damage to cereal crops. Recent researches have shown that the V153H mutant ZHD increased the specific activity against α-ZOL, but decreased its specific activity to β-ZOL. To understand whyV153H mutation showed catalytic specificity for α-ZOL, four molecular dynamics simulations combining with protein network analysis for wild type ZHD α-ZOL, ZHD β-ZOL, V153H α-ZOL, and V153H β-ZOL complexes were performed using Gromacs software. Our theoretical results indicated that the V153H mutant could cause a conformational switch at the cap domain (residues Gly161–Thr190) to affect the relative position catalytic residue (H242). Protein network analysis illustrated that the V153H mutation enhanced the communication with the whole protein and residues with high betweenness in the four complexes, which were primarily assembled in the cap domain and residues Met241 to Tyr245 regions. In addition, the existence of α-ZOL binding to V153H mutation enlarged the distance from the O_AE_ atom in α-ZOL to the NE2 atom in His242, which prompted the side chain of H242 to the position with catalytic activity, thereby increasing the activity of V153H on the α-ZOL. Furthermore, α-ZOL could easily form a right attack angle and attack distance in the ZHD and α-ZOL complex to guarantee catalytic reaction. The alanine scanning results indicated that modifications of the residues in the cap domain produced significant changes in the binding affinity for α-ZOL and β-ZOL. Our results may provide useful theoretical evidence for the mechanism underlying the catalytic specificity of ZHD.

## 1. Introduction

**Figure 1:**
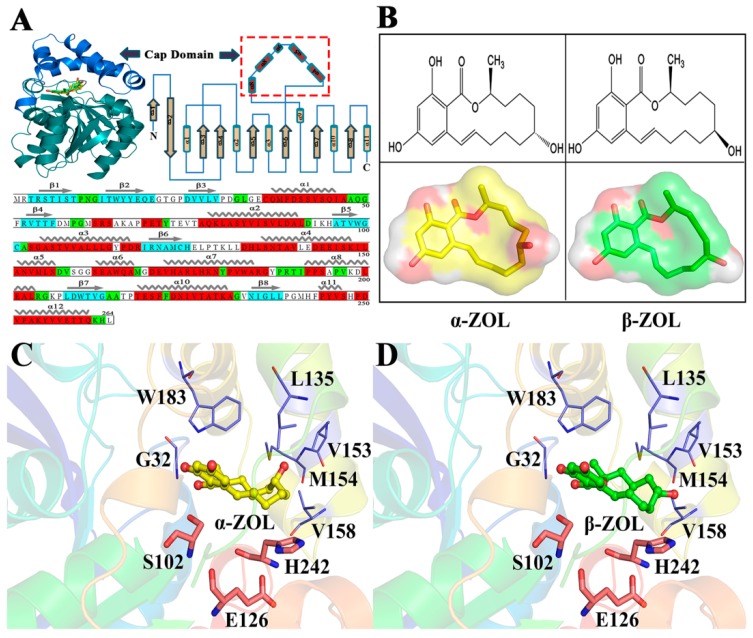
Structural organization of ZHD (zearalenone hydrolase) and coordination of the bound product analog in the active site of ZHD. (**A**) Domain composition of the ZHD monomer (upper left), topology diagram of ZHD (upper right), and structure sequence (bottom). The core domain is shown in green-cyan and the cap domain is shown in blue. The cap domain inserted in between β6 and α9. (**B**) Chemical structure of α-ZOL (α-zearalenol) and β-ZOL. (**C**,**D**) Binding pocket for α-ZOL and β-ZOL in ZHD. α-ZOL and β-ZOL are represented by yellow (**C**) and green (**D**) stick balls, respectively. The residues that surround the substrate are shown as purple sticks. Catalytic residues (Ser102, Glu126, and His242) are highlighted as heavy sticks.

Zearalenone (ZEN)—a resorcylic acid lactone with estrogenic activity that allows it to bind to and activate estrogen receptors—is a secondary metabolite of *Fusarium* species and is widely detected in ‘musty’ grains [1]. Exposure to ZEN-contaminated food or animal feeds may lead to the disruption of reproductive and endocrine systems, thereby causing huge economic losses in domestic animal farming, and posing a serious threat to human health [2,3,4,5]. Hence, the development of detoxifying strategies against ZEN is a key target in the animal feed and food industries. Zearalenol (ZOL) is the major natural derivative isolated from ZEN. Compared to ZEN, ZOLs are similar in form, but distinct in the functional group on C6′, which isoforms, α-ZOL, and β-ZOL (see Figure 1B). According to previous research, the α-ZOL exhibits higher toxicity than ZEN [2]. On the other hand, β-ZOL only exhibits half the toxicity of ZEN [3].

ZHD (zearalenone hydrolase) has demonstrated potential as a treatment for ZEN contamination that will not result in damage to cereal crops [6,7,8]. The amino acid structure sequence is presented in Figure 1A. Structures of apo ZHD and some enzyme-substrate complexes have recently been reported [4,5,9,10]. ZHD consists of a cap domain (Figure 1A, upper left blue) and a core domain (Figure 1A, upper left green cyan). As the upper right of Figure 1A shows, the cap domain is made up of five helixes (α helix4 to α helix9). The core domain of ZHD (Ser5–Pro128; Pro196–Leu264) has six helixes and eight β-sheets, and β2 is twice the length of other antiparallel strands. (Figure 1A top). The active site and substrate-binding pockets are located at the interface between the core and cap domains. These structures indicate the atypical catalytic triad consisting of Ser102–His242–Glu126 in an α-ZOL and β-ZOL binding site that is enclosed by the α/β-hydrolase fold and a helical cap domain. Close inspection of the substrate-binding site in ZHD (Figure 1C,D) reveals that the substrate is surrounded by Gly32, Trp183, Lue135, Val153, Met154, Val158, Ser102, His242, and Glu126. The triad of catalytic residues (Ser102–His242–Glu126) is located directly below the substrate.

In 2016, Guo et al. reported that the V153H mutant increased the specific activity against α-ZOL and decreased its specific activity to β-ZOL [4]. The work successfully improved ZHD activity toward the more toxic α-ZOL compound, which will be interesting for structure-based engineering and will show great potential chances in further industrial applications. However, we still do not understand the conformational changes between the wild-type (WT) and V153H mutants with substrate binding.

To understand the molecular mechanism of the substrate binding to WT and the V153H mutant, we considered chemical details and molecular dynamic (MD) simulations [11,12,13] for the intrinsic complexity of WT and V153H ZHD mutant and the substrate complex. The biological meaning of these findings are discussed as follows.

## 2. Results and Discursion

### 2.1. Structural Stability and Dynamics Properties of Four Complexes

**Figure 2:**
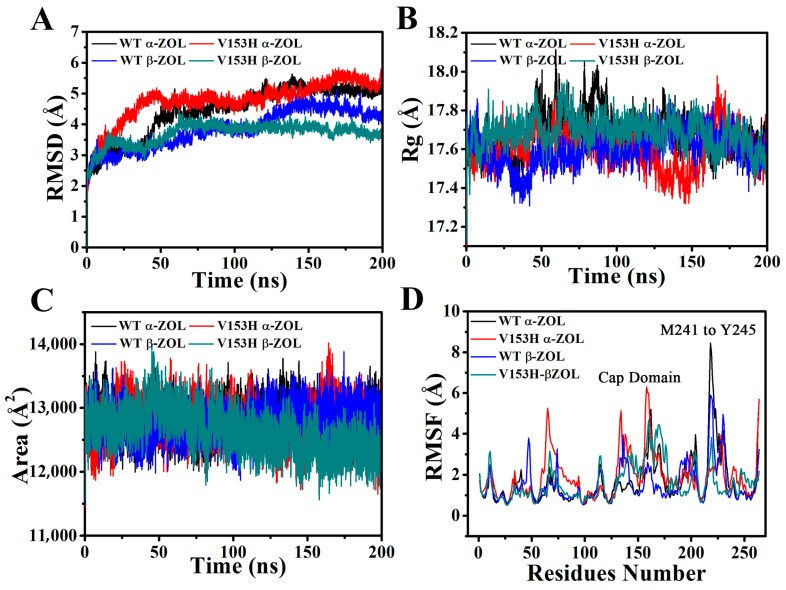
Stability analysis for WT α-ZOL (wild type α-ZOL), V153H α-ZOL, WT β-ZOL, and V153H β-ZOL. (**A**) The root-mean-square deviation (RMSD), (**B**) radius of gyration (Rg), (**C**) average solvent-accessible surface area (SASA), and (**D**) root mean square fluctuation (RMSF) for WT α-ZOL (black), V153H α-ZOL (red), WT β-ZOL (blue), and V153H β-ZOL (green–cyan) during 200 ns MD (molecular dynamic) simulations.

The 200 ns MD simulations were conducted for WT α-ZOL, V153H α-ZOL, WT β-ZOL, and V153H β-ZOL. Figure 2A showed the root-mean-square deviation (RMSD) values of backbone atoms for the four simulated structures. The RMSD versus simulation time was calculated to evaluate the stability of complexes during the simulations [14,15,16]. Our results indicated that the four complex systems that achieved equilibrium after 10 ns of simulation. The average fluctuations represented by the RMSD for the WT α-ZOL, V153H α-ZOL, WT β-ZOL, and V153H β-ZOL were 4.65 ± 0.09, 4.54 ± 0.11, 3.89 ± 0.09, and 3.56 ± 0.11 Å, respectively. The decrease of RMSD in the V153H mutant suggested that the stability of the protein structure was enhanced upon the binding of the substrate. The radius of gyration (Rg) of WT and mutant structures (Figure 2B) were calculated to gain insight into the overall dimensions of the proteins. It can be seen that, compared to β-ZOL, the α-ZOL could increase the fluctuations of Rg of ZHD, which suggested that stability fluctuation is not only a result of mutation, but also of binding to different substrates. The average solvent-accessible surface area (SASA) of ZHD for the four complexes showed a steady trend and the values of SASA were very similar, which indicated that the active mutants underwent expansion. This finding suggested that the activating mutations may experience stability loss due to small local unfolding.

The flexibility of each residue was assessed by its root mean square fluctuation (RMSF). Figure 2D showed the RMSF values of four systems calculated from 200 ns trajectories. For the four systems, the cap domain (residues Lys130–Arg175) and the C-terminal of residues Ala215–Lys230 exhibited large fluctuation values. For the WT α-ZOL system, the RMSF values of the cap domain were lower than those of the mutant systems. For V153H α-ZOL systems (red line in Figure 2D), most of the residues showed more obvious fluctuations than other systems. To confirm the results, the B-factor for four complexes were calculated and the results are shown in Figure S1. As we can see, the flexibility trend of each residue was consistent with the above RMSF analysis.

### 2.2. Protein Network Analysis of the ZHD Structures: V153H Mutation Enhances Communication between Different Residues

**Figure 3:**
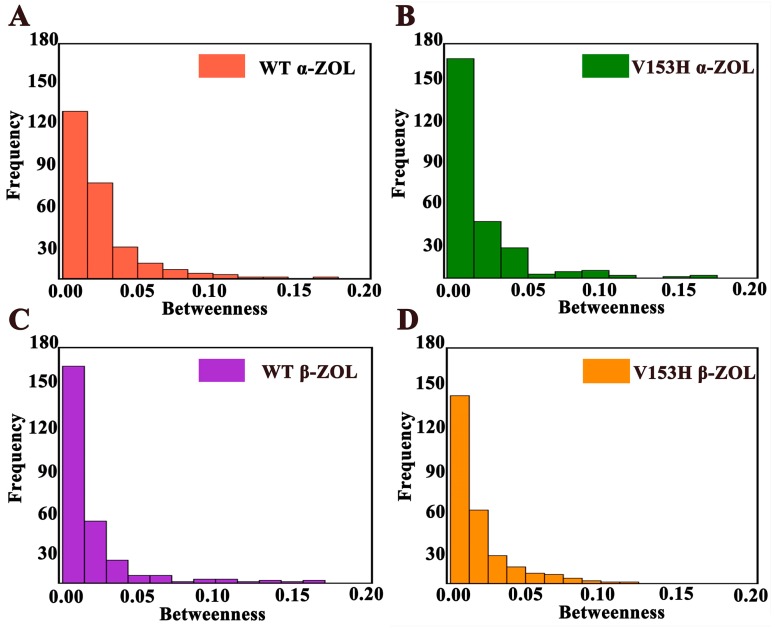
The frequency distributions of the network centrality parameters in ZHD structures. The frequency distributions of the betweenness values are shown in WT and V153H ZHD for WT α-ZOL (**A**), V153H α-ZOL (**B**), WT β-ZOL (**C**), and V153H β-ZOL (**D**).

To further study the effect of mutation on protein structure, we combined a structure-based network analysis with MD simulations of WT and V153H mutants to analyze the change of residue interaction communities. The weighted graphs and common measures of node centrality (degree and betweenness) were prepared to characterize local and global connectivity in the residue interaction networks. Both the topology-based residue connectivity and the contact maps of residue cross-correlations were used to construct the networks. For stable interaction communities, it was conducted to detective the reorganization of residue connection networks and structurally conserved communities with different functional forms of ZHD. The distribution of local residue hubs during the simulations in the conformational ensembles was obtained, and the results were shown in Figure S2. The number of hub nodes in the four systems mainly ranged from six to eight. Unlike in WT α-ZOL and WT β-ZOL, the degree distributions in V153H α-ZOL and V153H β-ZOL were reminiscent of Poisson-like distributions with a large number of highly-connected hubs. The highly-connected local hubs in the V153H structures corresponded to the total residues characterized due to the high residue centrality and global connectivity. In a word, V153H enhanced the level of co-operativity of proteins. To confirm the results, the frequency distribution graphs of the betweenness values in WT and V153H were constructed. Similar to other protein structure studies, the results of ZHD structures interaction networks exhibited a Poisson-like centrality distribution (Figure 3) and a small-world network organization. The center profile in the V153H mutant showed a sharper decay and followed by a longer distribution tails, which departed from the random graph model. The thermal stability, specificity of catalysis, and ligand binding of enzymes could be significantly influenced through specific functional requirements. In order to analyze the global centrality, structural stability and functional significance of key residues for ZHD, the spatial distributions of key residues and the network centrality profiles in WT and V153H ZHD were prepared. The residues with a higher betweenness in the WT ZHD were primarily assembled in the cap domain and residues of the Met241 to Tyr245 regions (Figure S3A,B), but were more uniformly distributed in the V153H ZHD (Figure S3C,D). In the four structures, the betweenness profile revealed the noticeable peaks corresponding to important functional residues. These results were consistent with the RMSF and B-Factor analyses. In summary, the V153H mutation could influence the local and global connectivity of the entire protein. The cap domain and residues Met241 to Tyr245 region were active and important in the protein interaction network.

### 2.3. Comparison of the Conformational Changes of WT and V153H ZHD

To explore the influence of mutations on ZHD conformation, the define secondary structure of proteins (DSSP) for the four systems were prepared and the results were exhibited in Figure S4. Compared with wide type ZHD (Figure S4A,B), V153H mutants (Figure S4C,D) could result in a conformational switch at the cap domain (residues Gly161–Thr190). In the V153H mutant, the conformation of residues Gly161 to Thr190 in the cap domain transformed to turn from α helix at 53 ns and continued to 175 ns. Similarly, the conformation of residues Gly161 to Thr190 in V153H β-ZOL system became turned at 49 ns and contained to the simulation end. The turn content in the cap domain of V153H-α-ZOL and V153H-β-ZOL mutants exceeded 60% during the simulations (Table 1). To further confirmed the above results, the structures at different simulation times were extracted according to the trajectories from four systems (Figure S4). The α helix in the cap domain disappeared from V153H α-ZOL at 105.45 ns (Figure S4B red cartoon). Moreover, the α helix disappeared from V153H β-ZOL at 105.45 ns (Figure S4D red cartoon). However, WT ZHD did not make such a conformational change.

**Figure 4:**
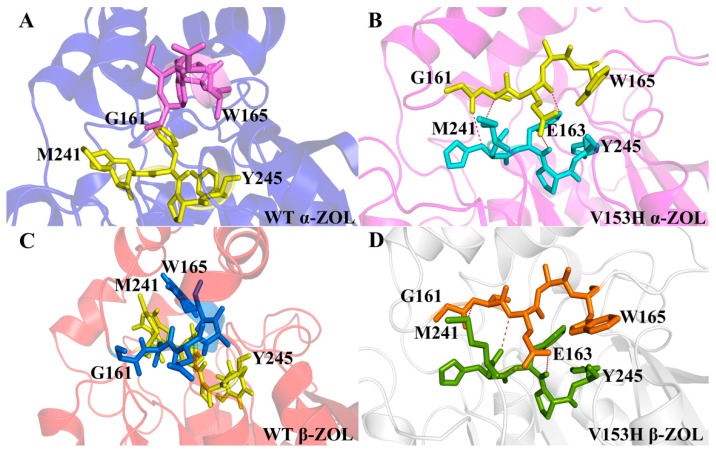
The intermolecular hydrogen bonds network from residues Gly161 to Trp165 and residues Met241 to Tyr245 for the four complexes. (**A**–**D**) The hydrogen bonds for residues Gly161 to Trp165 and Met241 to Tyr245. The accepter and donor residue of hydrogen bonds were shown as sticks. The hydrogen bonds were showed as red dash lines.

To explain the cause of change in the cap domain regulated by the activity of protein, we investigated the hydrogen bonds network of the cap domain during 200 ns MD simulations in the four complex systems. The results were shown in Figure 4. As seen in the Figure 4, in the V153H mutant (Figure 4B,D), residues Gly161 to Trp165 had strong hydrogen bond interactions with residues Met241 to Tyr245. In more detail, Gly161, Glu163, and Trp165 made hydrogen bonds with Met241, His242, and Tyr245, respectively. Meanwhile, in WT α-ZOL (Figure 4A) and WT β-ZOL (Figure 4B), hydrogen bonds did not exist, which was accompanied by the disappearance of the α helix from the cap domain. The hydrogen bond occupations between residues Gly161 to Trp165 and residues Met241 to Tyr245 were shown in Table 2. The total occupations between these two groups showed a significant upward trend in V153H α-ZOL and V153H β-ZOL. This phenomenon indicated that the disappearance of the α helix in the cap domain could enhance the interactions with residues Met241 to Tyr245 through hydrogen bonds, which might directly affect the conformation and formation of hydrogen bonds in the active center.

### 2.4. V153H Mutation in WT α-ZOL Could Lead to Catalytic Center of Be an Active Conformation

**Figure 5:**
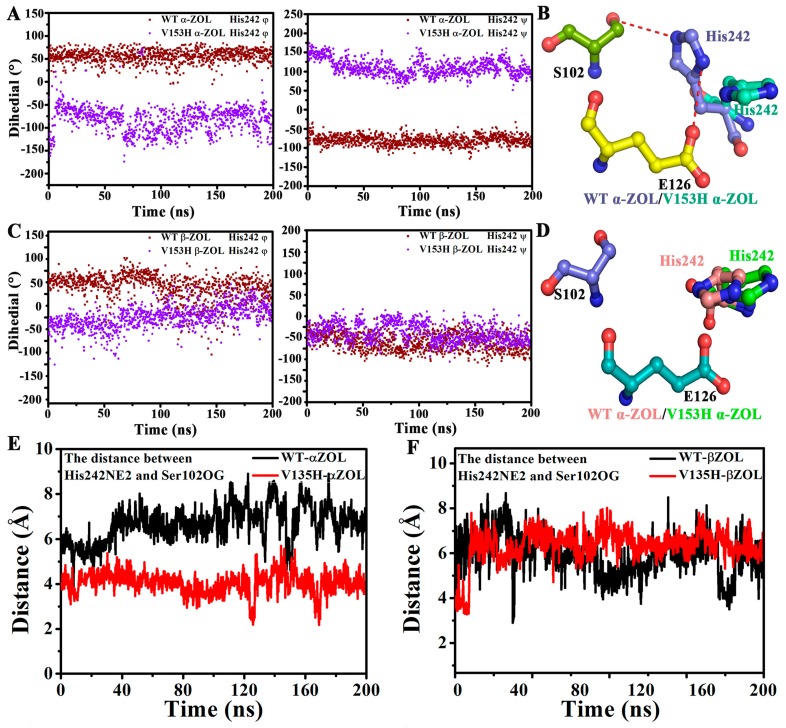
Comparison of the relative position of His242 in the triad catalytic center. (**A**,**C**) Comparison of the dihedrals (φ and ψ) of His242 in the WT α-ZOL, V153H α-ZOL, WT β-ZOL, and V153H β-ZOL systems. (**B**,**D**) The relative positions of Ser102, Glu126, and His242 in the WT α-ZOL, V153H α-ZOL, WT β-ZOL, and V153H β-ZOL systems. (**E**,**F**) The distance between His242:NE2 and Ser102:OG in the WT α-ZOL, V153H α-ZOL, WT β-ZOL, and V153H β-ZOL systems.

The catalytic center consists of Ser102–His242–Glu126. The relative position of His242 to the other two residues determines ZHD activation. In this simulation, we also analyzed the relative position of the His242 during the 200 ns simulation, and the results were shown in Figure 5. Figure 5A,C illustrated the dihedrals (φ and ψ) of His242 in the four systems. Compared with the other three systems, the V153H mutation in the V153H α-ZOL system caused the φ dihedrals of His242 to change from 50° to −50°, and the ψ dihedrals to change to 100°. The relative position of His242 in the triad catalytic center showed that the imidazole group of the His242 side chain in WT α-ZOL, WT β-ZOL, and V153H β-ZOL were perpendicular to the connecting line between the center mass of Ser102 and Glu126 (Figure 5B,D). On the contrary, the imidazole group in the V153H mutation in the V153H α-ZOL system (Figure 5B) was parallel to the connecting line, which helped to form a hydrogen-bonding network of Ser102–His242–Glu126. Afterwards, the distance from His242:NZ2 to Ser102:OG of the four complexes were detected. Figure 5E,F show that the distances from His242:NZ2 to Ser102:OG in WT α-ZOL, WT β-ZOL, and V153H β-ZOL increased by around seven Å, whereas it was only 3.7 Å in the V153H α-ZOL complex. The change in the relative conformation of His242 in the V153H α-ZOL complex decreased the distance between NZ2 of His242 and Ser102:OG, which helped facilitate the catalytic reaction.

**Figure 6:**
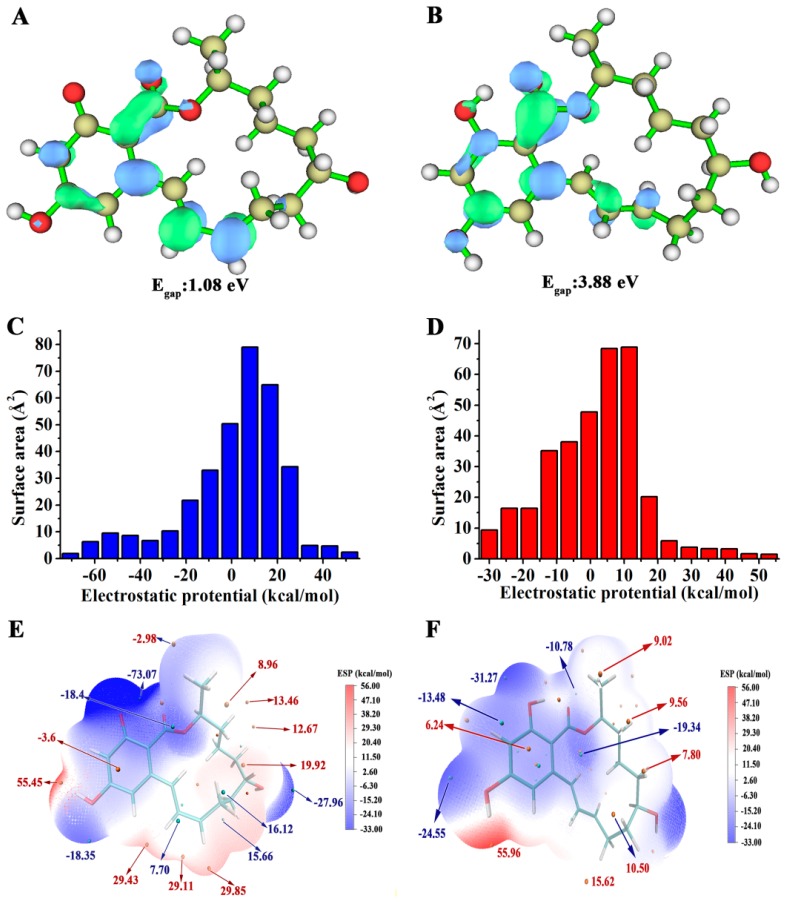
The LUMO orbits and electrostatic potential information of α-ZOL and β-ZOL. (**A**,**B**) LUMO orbits of α-ZOL and β-ZOL. (**C**–**F**) Surface area in each electrostatic potential (ESP) ranges on the vdW surface of (**C**) α-ZOL and (**D**) β-ZOL. ESP-mapped molecular vdW surface of (**D**) α-ZOL; and (**F**) β-ZOL.

Why then can V153H α-ZOL change the relative position of His242, yet the same effect is not achieved in V153H β-ZOL? This difference can be determined and influenced by the nature of α-ZOL and β-ZOL. Figure 6A,B showed the calculation of the LUMO orbital composition for α-ZOL and β-ZOL, representing the active part of the compound. Egap represented the energy difference between the HOMO and LUMO orbits, which depended on all of the coordinates and provided a more efficient sampling approach than a geometrical reaction coordinate to better reflect the activities of the compounds. Egap values showed the trend as follows: α-ZOL < β-ZOL, indicating that the electron transfer may occur more easily in α-ZOL than in β-ZOL. The electrostatic potential (ESP) result shows that the values were −73.07 to 55.45 (kcal·mol^−1^) for α-ZOL (Figure 6A,E) and −33.48 to 55.96 (kcal·mol^−1^) for β-ZOL (Figure 6B,F), which suggested that the ESP distribution on the vdW surface fluctuates more remarkably in α-ZOL than in β-ZOL. The ESP distribution of α-ZOL also covered greater scope than that of β-ZOL. The O atom of α-ZOL showed a more negative charge than that of β-ZOL and was useful for the attack of the hydroxyl group of Ser102. As shown in Figure S1, compared with α-ZOL, the O_AE_ atom in β-ZOL was closer to the His242 in the catalytic center. The distance from O_AE_ in ZOL to the NE2 in His242 were calculated, and the results were exhibited in Figure S5. The distance in both WT β-ZOL and V153H β-ZOL mutants were closer than in WT α-ZOL and V153H α-ZOL. Such a close contact might cause an interaction between the hydrophilic O_AE_ in β-ZOL and NE2 in His242 and inhibits the side chain of His242 to the position with catalytic activity.

**Figure 7:**
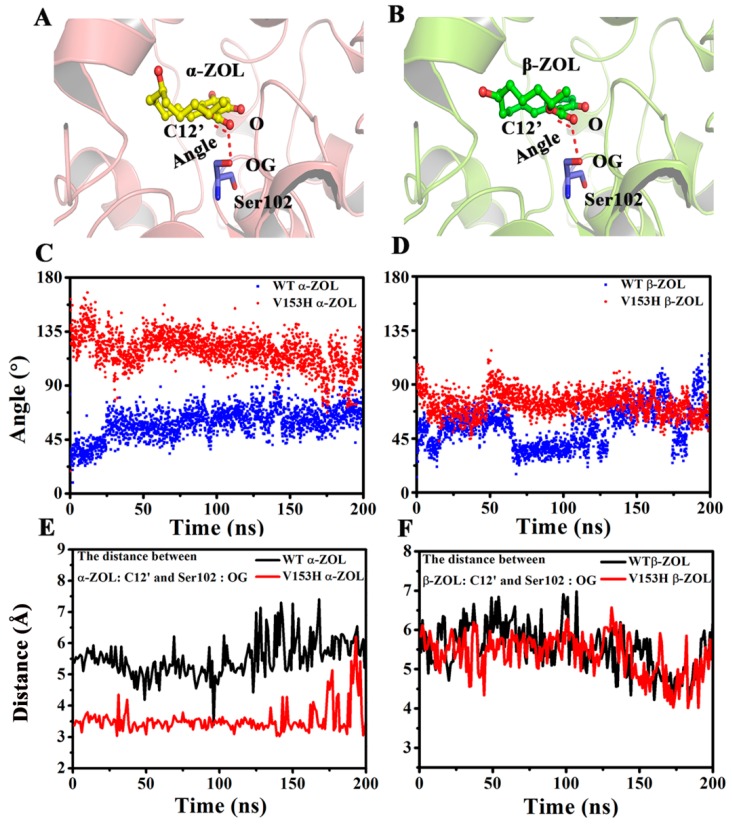
Comparison of the distance and angle of the affinity attacks of the four complexes during the 200 ns simulations. (**A**) The angle of C12′(α-ZOL)–O(α-ZOL)–OG(Ser102) for WT α-ZOL and V153H α-ZOL. (**B**) The angle of C12′(β-ZOL)–O(β-ZOL)–OG(Ser102) for WT β-ZOL and V153H β-ZOL. (**C**) The change in angle of (**A**) during the 200 ns simulations. (**D**) The change in the angle of (**B**) during the 200 ns simulations. (**E**) The distance between α-ZOL:C12′ and Ser102:OG in WT α-ZOL and V153H α-ZOL. (**F**) The distance between β-ZOL:C12′ and Ser102:OG in WT β-ZOL and V153H β-ZOL.

As a typical α/β hydrolase, ZHD is necessary to form a correct catalytic angle and distance with the substrate during catalytic reaction. Figure 7A,B showed the catalytic angle of ZHD and α-ZOL and β-ZOL. This angle must be between 110 ± 20°, guaranteeing the occurrence of a catalytic reaction. The angle of C12(ZOL)–O(ZOL)–OG(Ser102) for the WT and V153H mutation was detected, and the results were exhibited in Figure 7C,D. The angle was 75° for WT α-ZOL and β-ZOL. Meanwhile, V153H in V153H α-ZOL caused the increase of the angle to around 110° (Figure 7C), which can increase the activity of ZHD. Furthermore, the distance between ZOL:C12′ and Ser102:OG in the simulation was calculated (Figure 7D,E). The WT α-ZOL complex decreased the distance between ZOL:C12′ and Ser102:OG to facilitate the catalytic reaction. This difference may be the reason why the V153H mutation can increase activity against α-ZOL but not in β-ZOL.

### 2.5. Dominant Domain Motions

The correlation matrix obtained through principal component analysis (PCA) can improve our understanding of protein regions that present intense relevant conformational changes and can help clarify the mutation-induced dynamic motions of ZHD. The covariance matrix maps of four complexes during the 200 MD simulations were illustrated in Figure S7, and antiharmonic and large-scale motions were highlighted by diagonalizing the matrix. The positive regions marked in cyan indicated the strongly-correlated motions of the residues. The negative regions colored in pink were associated with anti-correlated movements. The diagonal was relatively highly correlated because it represented the variance of a residue with itself. In general, the values of −0.1 and 0.1 indicated the normal range of motion. Compare with the substrate binding, the V153H mutant had stronger positive correlations and negative movements than the WT ZHD. The matrix maps of the four systems indicated that significant motions, whether correlated or uncorrelated, mainly occurred in the region of residues Lys130 to Thr190 (cap domain) and residues Met241 to Tyr245. The V153H mutant showed significant negative correlation, as shown by how the pink area increased in the map in Figure S7B,D. These results were represented by the expansion of the cyan area in the activation segment shown in the matrix maps and were consistent with the fluctuations of RMSF and B-factor. In V153H mutant, residues Met1 to Gly50 showed strongly and negatively-correlated movements in the cap domain. In addition, residues Leu200 to Ser220 exhibited strongly and negatively-correlated movements with residues Val75 to Ala90 in V153H α-ZOL. The cross-correlation results suggested that the internal motions of ZHD were significantly affected by the mutation.

**Figure 8:**
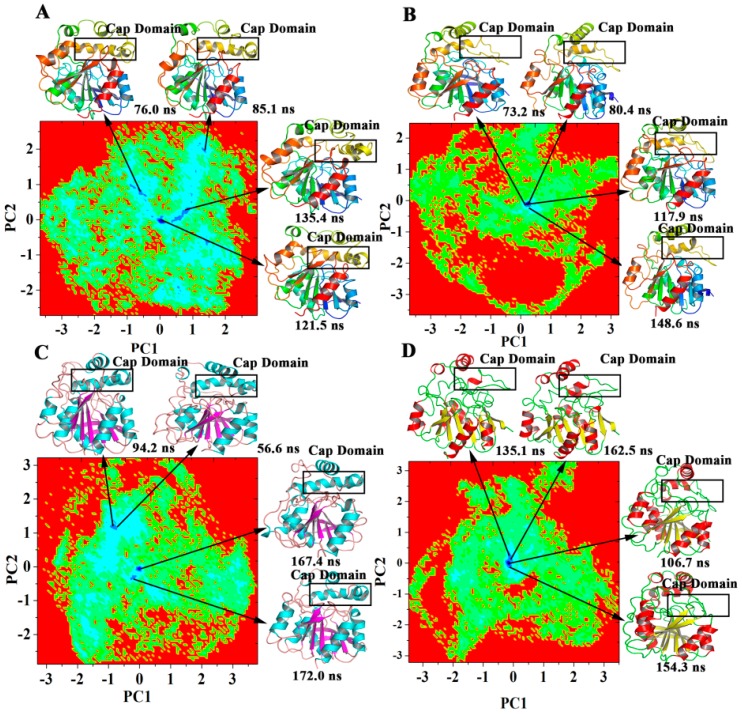
Analysis of the FEL of WT and V153H ZHD proteins. (**A**–**D**) The FEL map of WT α-ZOL, V153H α-ZOL, WT β-ZOL, and V153H β-ZOL systems. Blue represents the conformation of ZHD proteins with one global minimum. High numbers of blue regions indicate that more than one local minimum has been achieved in the simulation, and the intensity of the blue color is directly proportional to the minimum energy state of the protein. The depth of the three-dimensional energy landscape indicates the value of the minimum free energy. High depths indicate stable structures. Four representative structures of the most populated clusters in each system.

PCA and free-energy landscape (FEL) were independently performed on each trajectory of the four systems to reveal the precise structural differences between the WT and the V153H mutant. The extreme projections for PC1 and PC2 during 200 ns of MD simulation were visualized (Figure 8), showing that the WT ZHD had lower energy than the V153H mutant. The conformational transitions of the activation segment and pro-rich loop were observed in different regions of the FEL, as indicated by how the low-energy area is larger in Figure 8A,B. The representative structures of ZHD in the four systems were compared, and the results were shown in Figure 8. The cap domain in the low-energy structure of WT ZHD exhibited helix conformation. Meanwhile, the cap domain became turned in the low-energy structure of V153H ZHD. This suggested that the V153H mutation led to changes in conformation in the cap domain as a stable process. To detect the proportion of the lowest-energy conformation for the eight complexes in the entire simulation, the percentage of each PC in the eight simulation complexes were analyzed on the basis of their corresponding eigenvalues (Table 3). The sum percentage of PC1 and PC2 in the four systems reached more than 40%, which ensured the reliability of the results. Then, the contribution of each residue to the first two PCs was examined, as shown in Figure S6. Intense spikes were found at the positions of the cap domain and the residues Met241 to Tyr245 for the four systems, suggesting that these two regions provided the major contributions to the motions of the PC. This result is consistent with the RMSF values.

### 2.6. Computational Mutagenesis of Active Site Residues

**Figure 9:**
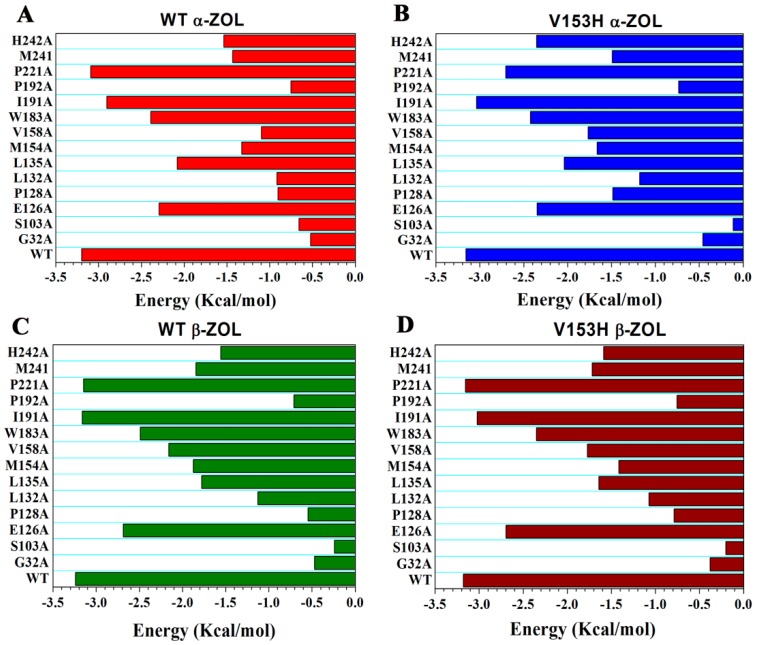
Computational alanine scanning of the binding site residues in the WT and V153H ZHD complexes. The analysis was performed using the FoldX approach applied to conformational ensembles obtained from 200 ns MD simulations. Binding free energies and alanine scanning of the binding site residues were shown for WT α-ZOL (**A**), V153H α-ZOL (**B**), WT β-ZOL (**C**), and V153H β-ZOL (**D**). Energetic binding hotspots correspond to residues for which alanine scanning resulted in a significant decrease of the binding free energy.

MD trajectories were then employed to carry out binding free energy calculations and to perform a systematic alanine scanning of the binding site residues in the WT and V153H mutant complexes (Figure 9). The main objective of this analysis was to identify energetic hot spots that contribute to the elucidation of the roles of individual residues on the binding of the substrate. The same change trend was observed in the distribution of the energetic hotspots for the WT α-ZOL and V153Hα-ZOL (Figure 9A,B). For WT α-ZOL, the primary interacting residues that contribute significantly to binding affinity included Gly32, Ser103, Pro128, Lue132, and Pro192 (Figure 9A). Interestingly, alanine modifications of those residues produced minor changes in the binding affinity profile of α-ZOL. Indeed, the binding free energy changes upon mutations of Tyr183, Ile191, and Pro221 were small. The results of alanine scanning the V153H α-ZOL revealed the energetic hot spots corresponding to residues Gly32, Ser103, and Pro192 (Figure 9B). The same trend was found in WT β-ZOL and V153H β-ZOL (Figure 9C,D). These results suggested that the residues located at the cap domain played important roles in binding with both α-ZOL and β-ZOL.

## 3. Materials and Methods

### 3.1. Structure Preparation

The initial structure of ZHD in the MD simulations for four complex structures, namely, WT ZHD/α-ZOL (PDB ID 5IE4), WT ZHD/β-ZOL (PDB ID 5IE6), V153H ZHD/α-ZOL (PDB ID 5IE5), and V153H ZHD/β-ZOL (PDB ID 5IE7) [4], were taken from the Protein Data Bank independently. To keep the proteins in an active state, the Ala102 in four structures were mutated to Ser102 by using Pymol 4.5.0. The geometries of α-ZOL and β-ZOL were fully optimized using the density functional theory, with the B3LYP at 6-31G* level set with Gaussian 09 [17].

### 3.2. Molecular Dynamics Simulations

All the molecular dynamics simulations were prepared with Gromacs 5.1.4 package and the Gromos 53A6 force field [18] was applied to describe the protein and the ligand. The parameterization of the ligand was performed using the PRODRG2.5 server [19]. All complex systems were subjected to MD simulation in a periodic boundary box with the SPC water model [20]. Sodium and chloride ions were added toneutralize the simulated systems and to ensure an ionconcentration of 150 mM ionic strength. Energy minimization was performed through the steepest descent method. The energy-minimized structure was allowed to reach an initial structure of equilibration. Subsequently, 100 ps NVT (Berendsen temperature [21] coupling with constant particle number, volume, and temperature) and 100 ps NPT (Parrinello–Rahman pressure coupling with constant particle number, pressure, and temperature) [22] were used to keep the system in a stable environment (300 K, 1 bar). For first 500 ps, the temperature increased to 500 from 0 K at constant rate of 1 K/ps. Then it decreased to 300 K at the same speed. The MD simulation for NPT were run 500 ps at 300 K ensemble with a time step of 2 fs, which kept the temperature (300 K) and pressure (1 atm) through a Langevin thermostat [23] and a Langevin piston barostat [24] method, respectively. Long-range electrostatic interactions were described using the Particle Mesh Ewald algorithm [17] with an interpolation order of 4, and a grid spacing of 1.6 Å, and van der Waals interactions were calculated with a cutoff of 14 Å. All bond lengths were constrained using the LINCS algorithm [25]. After all thermodynamic properties had stabilized, the molecular system was simulated for 200 ns with a time step of 2 fs, and the coordinates for all models were saved every 2 ps.

### 3.3. Computational Alanine Scanning

Computation alalanine scanning [26] was used to identify the function epitopes. In this study, it was calculated using FoldX approach [27,28] according to 10,000 snapshots of the WT and V153H mutant complexes trajectories during the 200 ns simulation to analyze the binding free energy for the four complexes. A graphical user interface was utilized for the computation alalanine scanning calculations [29], which was carried out as a plug in for the YASARA molecular graphics suite [30]. If a free energy change between the mutant and the WT proteins (the active residues) ΔΔG = ΔG (MT) − ΔG (WT) > 0, the mutation was considered destabilizing, whereas, when ΔΔG < 0, a spective mutation was considered as stabilizing. To consider the functionally important dynamic changes, we computed the average ΔΔG values using multiple samples (200−300) from the equilibrium ensembles using a modified FoldX protocol [31,32].

### 3.4. Protein Structure Network Analysis

The network analysis of the WT and V153H mutation structures was analyzed by using generated graphs, in which each residue was defined as a node. They were connected by edges corresponding to noncovalent interactions. The interaction between two residues *i* and *j* was measured as:

(1) $$I_{ij}\;=\;\frac{n_{ij}}{\sqrt{{(N}_{i\;}{\times \;N}_{j})}}\;\times \;100$$

where *n_ij_* represents the number of distinct atoms from residues *i* and *j*. The normalization factors for residues *i* and *j* are *N_i_* and *N_j_*, respectively. A protein structure graph of a desired interaction cut-off (*I_min_*) was constructed by forming an edge between any two residues with *I_ij_* greater than a cut-off (*I_min_*). For residue-based centrality analysis, any pair of residues were connected in the protein structure graph if *I_min_* = 2.5%.

A weighted network representation of the protein structure was adopted in this study, and the model incorporates both the residue cross-correlation fluctuation matrix and non-covalent connectivity of side chains in the construction of network graphs [33,34]. The Floyd−Warshall algorithm was used to determine the shortest paths between residues [35] that enumerated all short paths in the graph connecting each pair of residue nodes. Using the constructed protein structure networks, the residue-based betweenness parameter was prepared to construct the networks of protein structures. The sum of the fraction of shortest paths between all residue pairs that pass through redidue *i*, which was defined as the betweenness of residue:

(2) $$C_{b}\left(n_{i}\right)\;=\;\overset{N}{\underset{j<k}{\sum }}\;\frac{g_{jk}(i)}{g_{jk}}$$

where *g_jk_* represented the number of shortest geodesics paths that connect *j* and *k*, while the number of shortest paths between residues *j* and *k* passing through the node *n_i_* was *g_jk(i)_*. Residues that had high betweenness values need to get high occurrence in connecting all residue pairs in the shortest paths. Then the hub detection was conducted to analyze the interaction networks. The number of the interacting residues connected to a particular residue node was defined by the degree of a node. The node was identified as a hub if the number of the node was at least four. In this study, interactive analysis, 2D visualization, RINerator, and RINalyzer for the automatized generation were used for the residue interaction networks analysis [36].

### 3.5. Network Centrality Analysis

The global centrality measures for residue degree, closeness and betweenness were used for the analysis of constructed protein structure networks [37]. The sum of the edge weights between the consecutive nodes (*n_k_*, *n_l_*) represented the length of a path *d* (*n_i_*, *n_j_*) between distant nodes *n_i_* and *n_j_*:

(3) $$d\left(n_{i},n_{j}\right)=\underset{kl}{\sum }w\left(n_{k},n_{l}\right)$$

The Floyd–Warshall algorithm [32] compared all possible paths for the graph between each pair of residue nodes, which determine the shortest paths between two residues *d* (*n_i_*, *n_j_*). Firstly, the connected residue distance was considered to be one, and the path whose two distant residues were connected by intermediate residues with the smallest number, which identified the shortened path.

Python module Network was performed for network graph calculations [38]. The short paths included intermediate residues with sufficiently correlated (*C_ij_* = 0.5–1.0) was considered to select the shortest paths. Based on the edge weight of connecting residues’ tolerance threshold *C_ij_* = 0.5; *w_ij_* = −{log(−0.5)} = 0.69, an ensemble for sub optimal pathways, which connected the separated spatially sites and was defined.

A centrality measure of the local connectivity was the degree of a node in the interaction network. The number of residue *i* direct connection to other residues was the degree and was calculated as followed:

(4) $$d\left(n_{i}\right)=\overset{N}{\underset{j=1}{\sum }}a_{ij}$$

where *a_ij_* represented the element for adjacency matrix A, and the total number of nodes was *N* in the residue interaction network. The sum of the fraction of shortest paths between all the residues that interacted with residue *i* was the betweenness of the residue. The residue would have a higher values of betweenness if it had a high occurrence in the shortest paths that connected all residue pairs. The normalized betweenness for residue *i* can be calculated as follows:

(5) $$C_{b}\left(n_{i}\right)=\frac{1}{\left(N-1\right)\left(N-2\right)}\overset{N}{\underset{\begin{array}{c} j<k\\ j\neq i\neq k \end{array}}{\sum }}\frac{g_{ik\;}\left(i\right)}{g_{ik}}$$

where *g_jk_* represented the number of the shorted paths from residues *i* to *k*, and the fraction of the shortest paths that pass through residue *i* was *g_jk_*(*i*).

## 4. Conclusions

ZHD is α/β-hydrolase can detoxify α-ZOL and β-ZOL, demonstrating a potential role in reducing the contamination of ZOLs in cereal crops. In this study, WT α-ZOL, V153H α-ZOL, V153H β-ZOL, and WT β-ZOL complexes were prepared for molecular dynamic simulation to discover the mechanism that led to the V153H mutant possessing a much higher activity for α-ZOL than β-ZOL. Distribution of local residue hubs indicated that the degree distribution in V153H mutants was reminiscent of a Poisson-like distribution with a large number of highly-connected hubs. The betweenness analysis illustrated that the V153H mutation enhanced the communications between different residues. The MD results revealed that the V153H mutation could change the cap domain of ZHD from a helix to a turn, which enhanced its interaction with residues Met241–Tyr245 through hydrogen bonds to regulate the catalytic residue (His242) to an active conformation. The existence of α-ZOL enlarged the distance from the O_AE_ atom in α-ZOL to the N_E2_ atom in His242, which prompted the side chain of H242 to the position with catalytic activity. In addition, α-ZOL can form a reasonable attraction angle and distance with ZHD to help the catalytic reaction. The alanine scanning results indicated that Gly32, Ser103, Pro128, Lue132, and Pro192 played important roles in binding with the substrate instead of other residues. All these results explain why the V153H mutation has increased activity against α-ZOL rather than β-ZOL.

## Figures and Tables

**Table 1:** Probability of generating turn in cap domain (residues 160 to 175).

System	Cap Domain	Turn Occupancy
WT α-ZOL	G161 to R175	3.40%
V153H α-ZOL	G161 to R175	62.8%
WT β-ZOL	G161 to R175	4.60%
V153H β-ZOL	G161 to R175	84.1%

**Table 2:** Hydrogen bond occupancies between residues G161 to W165 and residues M241 to Y245 during MD simulations.

Hydrogen Bonds		WT α-ZOL	V153H α-ZOL	WT β-ZOL	V153H β-ZOL
Donor	Accepter				
Ser162:OG	Gly240:O	11.39	41.96		24.48
Ser162:OG	Gly240:C	12.74	30.59	21.36	35.61
Ser162:OG	His242:N	23.49	42.59	15.67	36.86
Ser162:OG	His242:CB		25.53	13.64	47.25
Ser162:N	Met241:O	16.38	33.52	22.52	36.61
Ser162:OG	Phe243:N	24.26	48.35	10.25	34.03
Ser162:OG	Met241:O		38.01		21.03

**Table 3:** Principle component probability during MD simulations.

Protein	Principle Component (PC)	Probility (%)
WT α-ZOL	PC1	43.52
	PC2	13.58
V153H α-ZOL	PC1	42.34
	PC2	14.93
WT β-ZOL	PC1	49.75
	PC2	12.43
V153H β-ZOL	PC1	47.18
	PC2	20.37

## Supplementary Materials

Supplementary materials can be found at http://www.mdpi.com/1422-0067/19/9/2808/s1.
