# Supplementary material for: Theoretical Study on Zearalenol Compounds Binding with Wild Type Zearalenone Hydrolase and V153H Mutant
Source: Int J Mol Sci. 2018 Sep 18;19(9):2808. doi: 10.3390/ijms19092808 (PMC6165071; doi:10.3390/ijms19092808)

Figure S1

Conformational dynamics of WT α-ZOL, V153H α-ZOL, WT β-ZOL, and V153H β-ZOL structures. (A-D) Computed B-factors obtained from MD simulations of WT α-ZOL (A), V153H α-ZOL (B), WT β-ZOL (C), and V153H β-ZOL (D). The cap domain (residues 130 to 190) is highlighted by blue the line and the core domain is colored red.

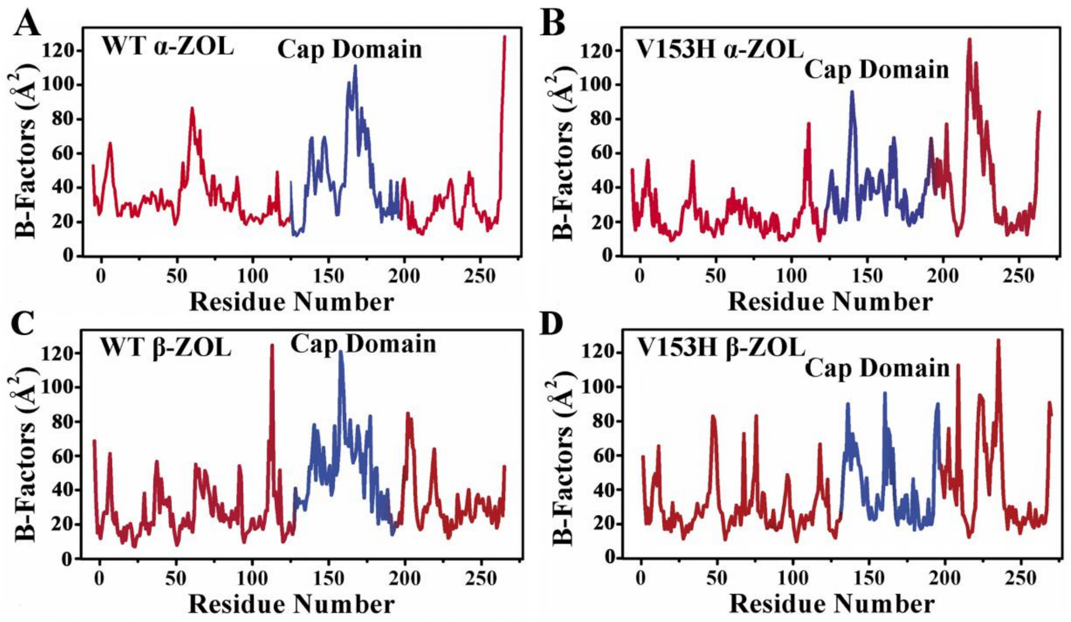

Figure S2

The network analysis of the ZHD structures. The degree distribution of residue hubs in the ZHD structures. The number of hub nodes as a function of the degree of a hub is shown for the WT α-ZOL, V153H α-ZOL, V153H α-ZOL, and WT β-ZOL.

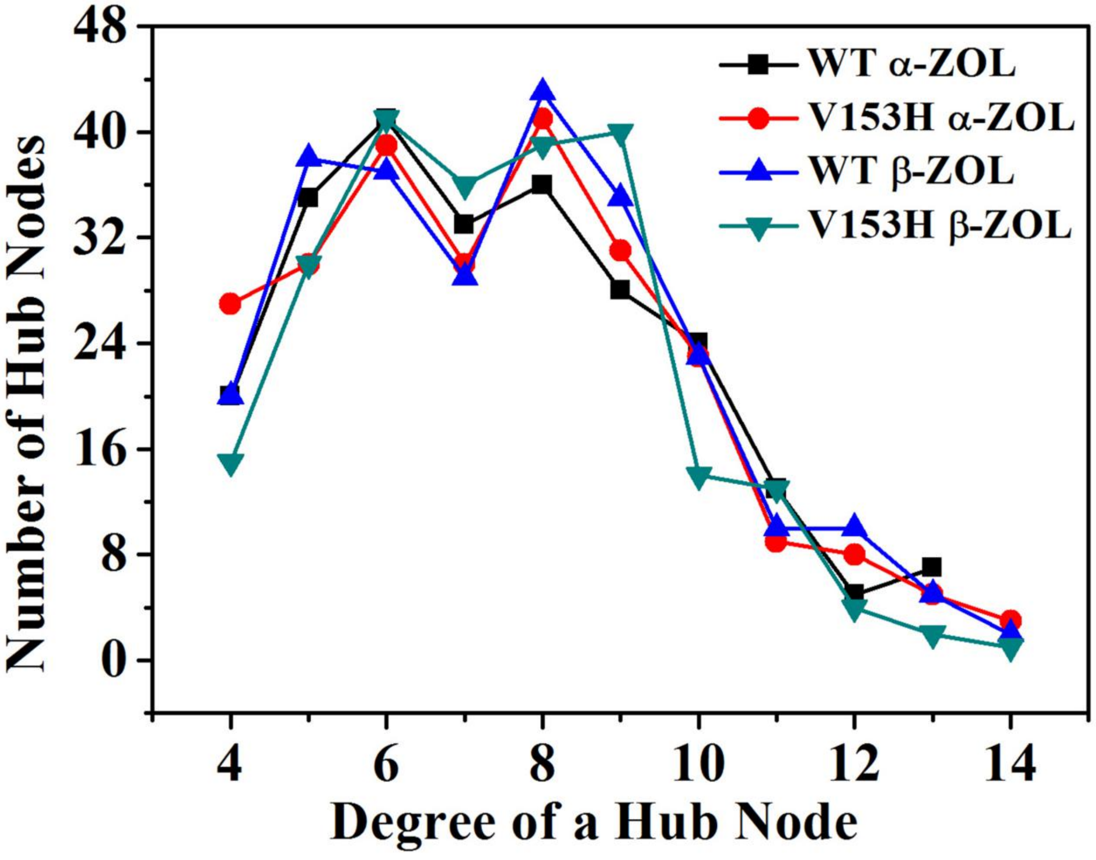

Figure S3

The residue-based betweenness profiles of the ZHD structures. Dynamics-based analysis of network centrality in the ZHD structures. The residue-based betweenness profiles are shown for the WT ZHD structures (A), V153H ZHD structures (B), the WT ZHD crystal structure (C), and the V153H ZHD (D) crystal structure. The peaks of the betweenness profiles corresponding to functionally important residues are indicated by arrows and annotated. The protein structures are shown in a cartoon representation. The functional residues of high centrality are shown on the surface. Structural positions of high centrality functional residues are indicated by arrows.

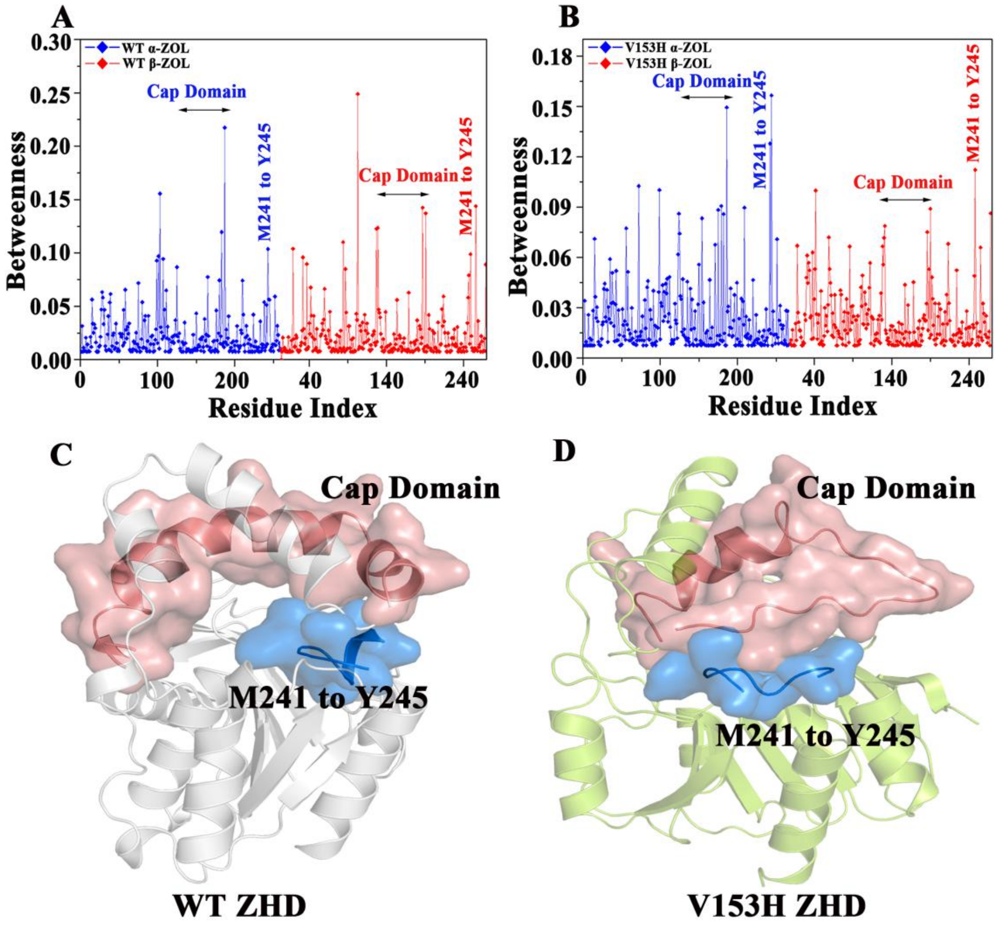

Figure S4

Dynamic changes in the secondary structure profiles of WT and ZHD mutant proteins throughout the simulation (A-D). The color bar represents different secondary structures as follows: helix (red), α-helix (pink), π-helix (purple pink), β-bridge (olive), β-bugle (yellow), turn (cyan), and coil (white). Structure of ZHD in WT α-ZOL (A), V153H α-ZOL (B), WT β-ZOL (C), and V153H β-ZOL (D) systems at 105.45 ns as depicted as cartoons. The residues Gly161 to Thr190 are highlighted by the deeper colors.

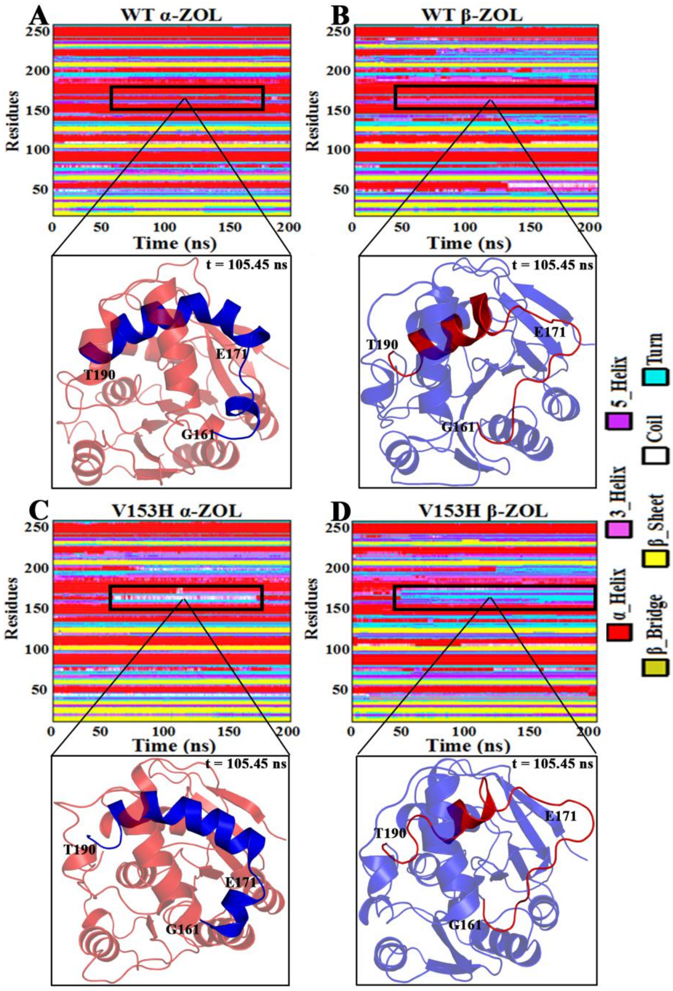

Figure S5

Comparison of the distance between His242:NE2 and ZOL:OAE four complexs during the simulation.

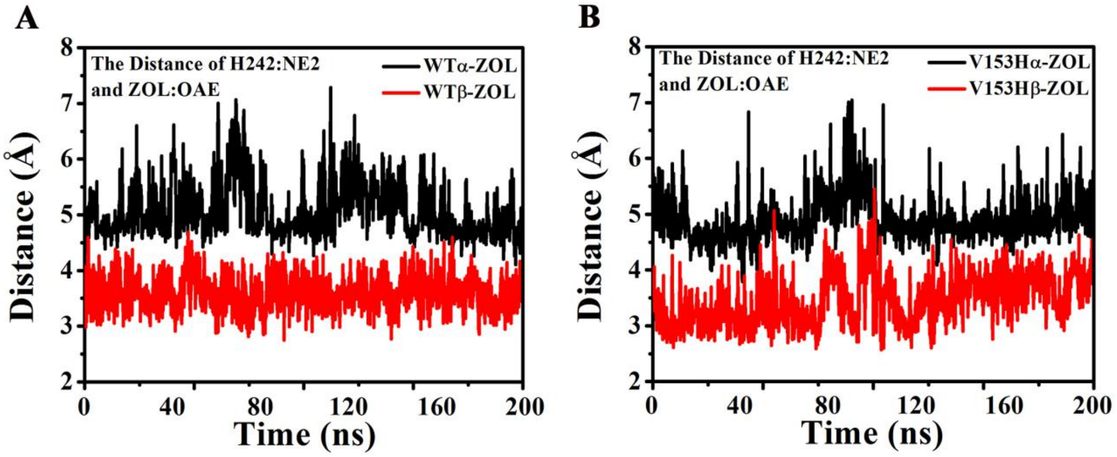

Figure S6

Contribution of residues to PCA in four systems. Black lines represent the contribution of PC1, and red lines represent the contribution of PC2.

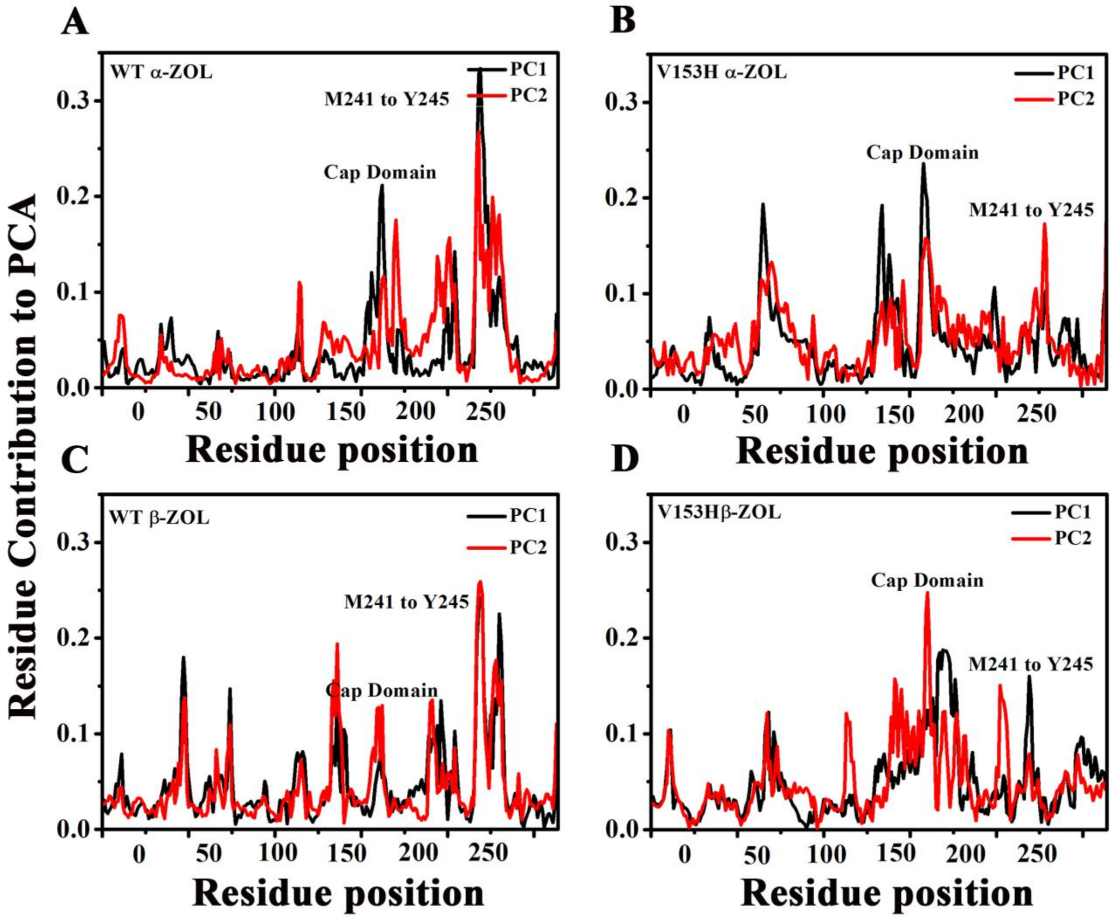

Figure S7

Cross-correlation matrix maps for WT and V153H ZHD proteins. Positive regions are marked in cyan and indicate strongly-correlated residue motions, whereas negative regions are colored in pink and are associated with anticorrelated movements. The positions of the activation segment and pro-rich loop are highlighted by boxes in each map.

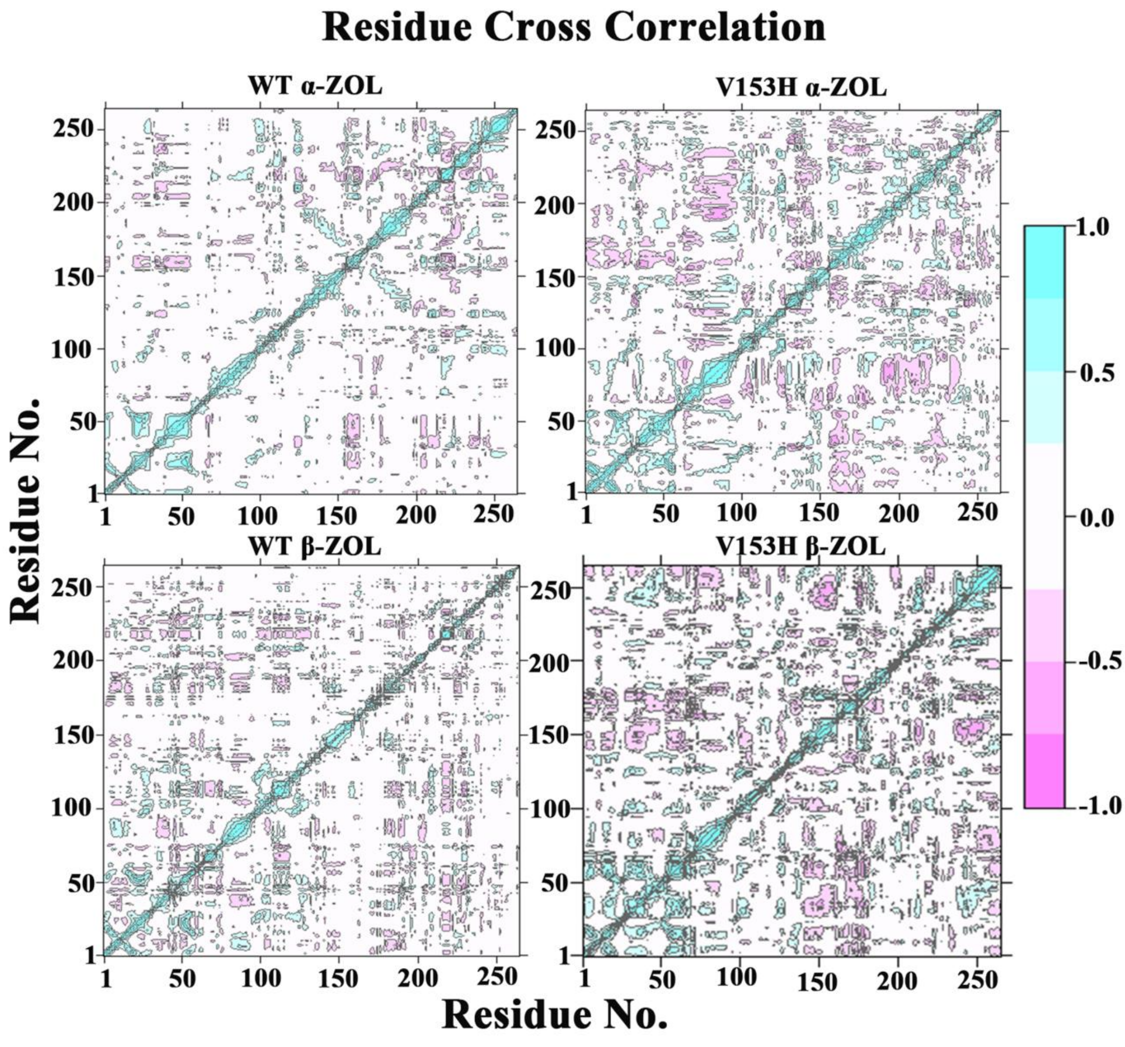

Supplement: Supplementary file 1 [file ijms-19-02808-s001.pdf]
